# Effectiveness of Herbal, Homeopathic and Conventional Dentifrices on Dental Caries – A Double-Blind Randomised Controlled Trial

**DOI:** 10.3290/j.ohpd.b4424883

**Published:** 2023-09-22

**Authors:** Shivashankar Kengadaran, Subhashree Rohinikumar, Divvi Anusha, Vyshiali Sundararajan, Katherina Barman

**Affiliations:** a Reader, Department of Public Health Dentistry, Indira Gandhi Institute of Dental Sciences, Sri Balaji Vidyapeeth, Pondicherry, India. Study design, participated in hypothesis formulation, Data collection, performed statistical analysis, wrote the manuscript, critically reviewed the manuscript.; b Reader, Department of Prosthodontics and Implantology, Saveetha Dental College and Hospitals, Saveetha Institute of Medical and Technical Sciences, Saveetha University, Chennai, Tamil Nadu, India. Study design, participated in hypothesis formulation, wrote the manuscript, critically reviewed the manuscript.; c Reader, Department of Public Health Dentistry, Indira Gandhi Institute of Dental Sciences, Sri Balaji Vidyapeeth, Pondicherry, India. Study design, participated in hypothesis formulation, performed statistical analysis, wrote the manuscript, critically reviewed the manuscript.; d Program Analyst, California Department of Public Health, Sacramento, CA, USA. Study design, critically reviewed the manuscript.; e Senior Lecturer, Department of Paediatric and Preventive Dentistry, Indira Gandhi Institute of Dental Sciences, Sri Balaji Vidyapeeth, Pondicherry, India. Study design, critically reviewed the manuscript.

**Keywords:** biofilm, dental decay, dental plaque, saliva, toothpastes

## Abstract

**Purpose:**

To compare the effectiveness of ayurvedic, homeopathic and conventional dentifrices on plaque and saliva in terms of cariogenic bacteria, salivary pH, and plaque pH.

**Materials and Methods:**

This double-blinded, parallel-group, randomised controlled trial was performed at Saveetha Dental College and Hospitals, Chennai, India. The participants comprised healthy adults possessing more than 20 permanent natural teeth and having a Decayed Missing and Filled Teeth (DMFT) score, plaque index score, and gingival index score less than or equal to 2. There were 3 intervention groups: 1: herbal dentifrice (Dabur Meswak); 2: homeopathic dentifrice (Gum Forte gel); 3: fluoride dentifrice (Colgate Total). The outcome measures were as follows: plaque and saliva samples were evaluated for pH; colony counts of *Streptococcus mutans* and *Lactobacillus* at baseline, 14 and 28 days of follow-up. One-way and repeated measures ANOVA, Wilcoxon signed-rank and Kruskal Wallis tests were used to compare the mean differences of plaque and salivary pH and plaque and salivary *S. mutans* and *Lactobacillus* counts at baseline, 14 and 28 days.

**Results:**

The mean *S. mutans* and *Lactobacillus* counts in plaque and saliva decreased statistically significantly in all treatment groups at the 28-day follow-up. Mean plaque pH was not statistically significantly different at the 14-day follow-up (p-value = 0.16). On the 28th day, group 1 (7.64 ± 0.20) showed the highest increase in plaque pH followed by group 2 (7.39 ± 0.25) and group 3 (7.27 ± 0.19), which was found to be statistically significant. No statistically significant difference in mean salivary pH was observed between the three groups at the different time points.

**Conclusion:**

This study reveals that the herbal dentifrice tested here was effective in reducing cariogenic bacterial count and increasing the plaque pH, thereby warranting the usage of the same.

Despite global improvement in oral health, certain oral diseases continue to be major public health problems, with dental caries and plaque-induced gingivitis being the most prevalent among them.^12^ Dental plaque, a microbial biofilm, is the primary aetiological factor leading to the initiation of gingivitis and dental caries. It is therefore important to prevent the formation and accumulation of plaque on the tooth surface by taking effective plaque-control measures.^18^

Self-performed mechanical plaque removal is a proven method of controlling plaque and gingival disease.^20^ However, toothbrushing and flossing can be difficult tasks and depend on the individual’s dexterity. Thus, many patients might not be able to completely remove plaque on all tooth surfaces. Furthermore, the high cost of oral care products in developing nations prevents the majority of the population from having access to adequate methods of maintaining a high level of oral hygiene. The inaccessibility of products in rural areas, low health literacy, and a lack of awareness could be other barriers.

Mechanical plaque control is a time-consuming procedure, and some individuals may lack the motivation for maintaining good oral hygiene. In an effort to enhance the efficacy of mechanical tooth-cleaning procedures, antimicrobial agents have been added to dentifrices.^6^ During the past decades, alternative medical treatment concepts have received significant attention. These concepts led to the development and marketing of oral health products with natural ingredients. A variety of dental products are currently being promoted as alternatives to the established oral hygiene products containing natural and herbal ingredients.^4^

*Salvadora persica* (miswak) has a centuries-long history in folk medicine. It has been used in oral hygiene, food, cosmetics, fuel, and even medicine. Many studies have reported its antimicrobial, antioxidant, analgesic, anti-inflammatory, sedative, wound-healing, and antitumor activities.^9,14,16,25^ The study by Al-Lafi et al^1^ reported an antibacterial effect of miswak sticks against aerobic and anaerobic oral bacteria. A systematic review showed the effectiveness of miswak against various types of oral bacteria implicated in caries or periodontal disease.^17^ The World Health Organization (WHO) has also recommended the use of *Salvadora persica* as an effective chemical plaque-control agent.

Homeopathy, on the other hand, is the second most popular system of medicines in the world, according to a report by WHO.^22^ According to Kengadaran et al,^14^ homeo-based products not only act as antimicrobial agents but also help in the development of the body’s immune system, thereby preventing the progression of dental caries. As the popularity of natural medicines and dentifrices continues to rise, dental professionals are in a position to provide information to patients about these products’ safety and efficacy. This can be difficult, owing to a lack of professional consensus on the subject.^8^ To date, insufficient clinical research on herbal and homeo-based dentifrices has been reported, in contrast to a plethora of such research for conventional fluoridated oral-care products. Hence, the present study was designed to investigate the effects of ayurvedic, homeopathic and conventional dentifrices on cariogenic bacteria, salivary pH, and plaque pH.

## Materials and Methods

### Study Design and Participants

In 2019, a four-week double-blind randomised controlled trial with a concurrent parallel design was conducted at a tertiary dental hospital in Chennai, India. Participants working at the tertiary dental hospital above the age of 18 years from non-health care backgrounds (i.e., office workers, computer operators and receptionists) were randomly selected and included in the study. A total of 90 participants were screened; after applying inclusion and exclusion criteria, 54 participants were enrolled in this clinical study.

To be included in the study, participants had to (i) be between the ages of 18 and 25 years, (ii) possess more than 20 permanent natural teeth, (iii) have a Decayed Missing and Filled Teeth (DMFT) score ≤ 2, and (iv) sign the written consent before beginning the study. Subjects who i) had taken antibiotics for the past 1 month, ii) were undergoing fixed orthodontic therapy, iii) had malocclusion, iv) exhibited hypersensitivity, v) were pregnant or lactating, vi) had developmental enamel and dentinal defects, vii) suffered from any systemic conditions, or viii) had reduced motor abilities were excluded from the study.

### Ethical Considerations

The study protocol was approved by the institutional ethics board. It complied with the guidelines for good clinical practice and the Helsinki Declaration of 1975, as revised in 2000. This trial was registered in the Clinical Trial Registry of India (registration number: CTRI/2017/02/007949). All the study participants were informed about the purpose, risks and benefits of the study, and they provided written informed consent to participate. This clinical was described according to the CONSORT recommendations from 2010.

### Sample Size Estimation

The sample size was estimated based on the results of a previous study conducted by Talha et al.^26^ Using a sampling software (G power version 3.1.9.2, Heinrich Heine University, Düsseldorf, Germany), the minimum sample size was calculated to be 15 patients per group (power 90% and α-error 5%). Anticipating a 10% dropout rate, the sample size was increased to 18 patients per group. All the clinical examinations were carried out by a single examiner who was calibrated before the study, and the intra-examiner reliability was assessed (Cronbach’s α = 0.91).

### Randomisation and Blinding

The eligible participants were block-randomised equally into three groups: group 1: herbal (ayurvedic) toothpaste (Dabur Meswak; Ghaziabad, India); group 2: homeopathic toothpaste (Gum Forte gel, Fourrts India Laboratories; Chennai, India); Group 3: conventional fluoridated, triclosan-containing toothpaste (Colgate Total, Colgate Palmolive India; Mumbai, India). Each group comprised 18 participants. The principal investigator was blinded to the sequencing of the block and allocation of groups. The SNOSE (sequentially numbered, opaque, sealed envelopes) method was implemented for allocation concealment. Intervention allocation was revealed only after the data analysis was completed. Toothpaste was provided to the participants in identical tubes to make them unaware of the type of toothpaste allocated to them. The labels were not revealed until the end of the study. All the three varieties of toothpaste used in the study are commercially available.

### Outcome Assessment

At baseline, 14 and 28 days, patients were examined at the clinical research centre at Saveetha Dental College and Hospitals. The examination was performed with a CPI probe in the same clinical room each time. At baseline and the subsequent visits, the following indicators were used to assess clinical efficacy: a) pH of saliva; b) pH of plaque; e) *Streptococcus mutant* colony count in plaque and saliva; and f) *Lactobacillus* colony count in plaque and saliva. Participants were instructed to refrain from brushing for about 12 h before the clinic visits. Eating, drinking, and smoking were also prohibited for 4 h before examinations.

### Plaque and Saliva pH

The subjects were instructed to spit 1.5 ml of unstimulated saliva into a sterile plastic container, which was immediately transported to the lab. Supragingival plaque was collected from the buccal surface of maxillary right first molar region using a sterile surface scaler and then stained with a disclosing solution. The collected plaque was pooled and placed into a tube containing 1 ml of sterile phosphate-buffered saline (PBS). The collected saliva and plaque samples were immediately transported to the Biomedical Research Unit and Laboratory Animal Centre (BRULAC), Saveetha Dental College and Hospitals. The saliva and plaque samples were mixed with 10 ml of distilled water, and a portable pH meter was used for measurements.

### Bacteria Colony Counting

1.5 ml of the collected saliva sample was diluted ten fold with normal saline. Lactobacillus MRS agar and Mitis salivarius (MS) agar were prepared by mixing 90 g of agar in 1000 ml of distilled water, followed by boiling the mixture to ensure complete dissolution. This solution was then autoclaved at 15 lbs pressure and 121°C temperature for 15 min. After cooling to 50-55°C, 1 ml of 0.1% potassium tellurite was added to this mixture and poured into Petri dishes. Once the MS agar plates were dry, saliva samples were inoculated onto the plates, which were incubated for 24 h at a temperature of 37°C. The collected plaque was pooled and placed into a tube containing 1 ml of sterile phosphate-buffered saline (PBS). It was then diluted ten-fold with normal saline and inoculated onto the plates. The colonies were counted using an automatic colony counter. The number of colony-forming units (CFU) per ml was converted to a log10 value and recorded in the pre-structured proforma.

### Periodontal Therapy

After recording baseline data, each participant underwent thorough scaling using ultrasonic instruments to remove supra-gingival plaque. Then every patient was provided with the assigned toothpaste and a similar adult soft-bristled toothbrush. The subjects were instructed to use the given soft-bristled toothbrush and anti-cavity toothpaste (pea-sized or covering half the size of the toothbrush) and brush their teeth twice daily for 2–3 min, following the modified Bass technique for a minimum of 2 min at least twice a day. Instructions regarding the brushing technique were printed as a pamphlet and distributed to the subjects.

### Statistical Analysis

Data were analysed using SPSS software (v. 20, IBM; Armonk, NY, USA). The distribution of the data was tested using the Kolmogorov-Smirnov and Shapiro-Wilk tests. Repeated measures ANOVA was used to compare the differences in pH of plaque and saliva within the groups. One-way ANOVA was used to estimate differences between the mean plaque pH, and salivary pH between the groups. Microbial colony counts were converted to log10 values and analysed using the Kruskal-Wallis and Friedman tests. A p-value <0.05 was considered statistically significant.

## Results

Out of 90 candidates screened, 54 participants (66.7% female and 33.7% male) took part in the trial, with 18 in each group. All the participants completed the trial with full participation. The mean age ( ± SD) of the study participants was 21.61 ( ± 2.14) years. Baseline characteristics of the study participants are presented in Table 1. There was no statistically significant difference found in baseline variables among the three groups after allocation (p > 0.05).

Plaque pH at baseline and day 14 did not differ statistically significantly between the groups. Similarly, salivary pH did not differ statistically significantly between the three groups at baseline, 14 and 28 days. The mean plaque pH on the 28th day was highest in group 1 (7.64 ± 0.20) followed by group 2 (7.39 ± 0.25) and group 3 (7.27 ± 0.19). The differences were found to be statistically significant (p < 0.05) (Table 2). Intragroup comparison showed a statistically significant increase in plaque pH in group 1. The salivary pHs of all three groups were statistically similar (Table 2).

**Table 1:** Baseline characteristics of the study participants

	Group 1	Group 2	Group 3	p-value
Age, mean ( ± SD)	21 (2.22)	21.78 (2.07)	22.06 (2.12)	0.317
Gender n (%)				
Male	3 (16.7)	9 (50)	6 (33.3)	0.105
Female	15 (83.3)	9 (50)	12 (66.7)	
DMFT mean ( ± SD)				
D	0.81 (0.01)	0.79 (0.02)	0.83 (0.01)	0.241
M	0.04 (0.01)	0.04 (0.01)	0.042 (0.02)	0.931
F	0.94 (0.05)	0.9 (0.05)	0.98 (0.06)	0.841

**Table 2:** Comparison of the mean pH of saliva and plaque at different time points

	Time point	Group 1	Group 2	Group 3	p-value
Plaque pH	Baseline	7.31 (0.5)	7.3 (0.28)	7.37 (0.27)	0.808
	14th day	7.5 (0.34)	7.38 (0.22)	7.3 (0.18)	0.16
	28th day	7.64 (0.2)	7.39 (0.25)	7.27 (0.19)	0.00*
	p-value	0.013*	0.392	0.323	
Salivary pH	Baseline	7.6 (0.28)	7.4 (0.17)	7.59 (0.17)	0.051
	14th day	7.59 (0.24)	7.58 (0.19)	7.56 (0.24)	0.581
	28th day	7.53 (0.19)	7.53 (0.27)	7.58 (0.22)	0.825
	p-value	0.785	0.621	0.808	

* Statistically significant.

Intergroup comparison revealed no statistically significant difference between groups in CFU of salivary and plaque microorganisms. The intragroup comparison showed a statistically significant reduction in the CFU of salivary and plaque microorganisms in all groups at different time points (Tables 3 and 4).

**Table 3:** Comparison of the median *Streptococcus mutans* and *Lactobacillus* in saliva at different time points

Parameter	Group	Baseline Median (IQR)	14th day Median (IQR)	28th day Median (IQR)	p-value
Salivary *Streptococcus mutans*	Group 1	4.98 (4.79)	4.61 (4.41)	4.33 (4.32)	0.001
	Group 2	4.97 (4.90)	4.49 (4.46)	4.08 (4.43)	0.003
	Group 3	4.95 (5.03)	4.36 (4.52)	3.90 (4.29)	0.001
	p-value	0.876	0.078	0.160	
Salivary *Lactobacillus*	Group 1	4.56 (4.45)	4.38 (4.23)	4.08 (4.11)	0.001
	Group 2	4.51 (4.57)	4.34 (4.52)	4.08 (4.24)	0.001
	Group 3	4.53 (4.23)	4.08 (3.90)	3.90 (3.70)	0.001
	p-value	0.820	0.070	0.313	

p < 0.05 was considered statistically significant.

**Table 4:** Comparison of the median *Streptococcus mutans* and *Lactobacillus* in plaque at different time points

Parameter	Group	Baseline Median (IQR)	14th day Median (IQR)	28th day Median (IQR)	p-value
Plaque *Streptococcus mutans*	Group 1	4.86 (4.99)	4.51(4.70)	4.34(4.56)	0.003
	Group 2	4.92 (5.08)	4.15(4.68)	3.70(4.28)	0.002
	Group 3	4.94 (5.08)	4.30(4.46)	4.10(4.30)	0.0001
	p-value	0.750	0.637	0.327	
Plaque *Lactobacillus*	Group 1	4.30 (4.66)	4.15(4.53)	3.81 (4.36)	0.008
	Group 2	3.60 (4.44)	3.30(4.08)	2.70 (3.81)	0.012
	Group 3	0.00 (4.87)	0.00 (4.51)	0.00 (4.23)	0.011
	p-value	0.698	0.354	0.370	

p < 0.05 was considered statistically significant.

## Discussion

Despite being a preventable disease, dental caries remains the most prevalent chronic disease in both children and adults world-wide.^21^ Incontrovertible evidence has shown fluoride interventions to be the most effective means of preventing caries and remineralising initial caries lesions. The fluoride dosage allowed in oral care products has been carefully limited to avoid the risk of fluorosis in children and toxicity at all ages.^3^

Furthermore, concerns regarding the increase in antibiotic resistance in microorganisms against triclosan,^2^ an active component added in some fluoridated dentifrices, have promoted interest in the therapeutic use of non-conventional or alternative dentifrices, which provided the impetus for this study in 2019, when Colgate Total produced in India still contained triclosan. Originally, the reason for including triclosan in toothpaste was that it could fight harmful bacteria in dental plaque, while also reducing the swelling associated with serious gum disease. However, it was found that triclosan is an endocrine-disrupting chemical, leading to problems with proper hormone function. This is the reason why triclosan has meanwhile been removed from most fluoride toothpastes, including those made in India.

In the current double-blind randomised controlled trial, oral prophylaxis was carried out in study subjects to standardise the initial oral hygiene levels and to ensure uniformity of oral hygiene status similar to previous studies.^5,19^ Although the home oral-hygiene practices of the study subjects were not supervised, compliance was noted by the investigator every week by observing the amount of dentifrice that was remaining.

Saliva plays an important role in maintaining the dentition’s integrity by buffering acids produced by cariogenic bacteria and protecting teeth from decay. Saliva has been conventionally used as a diagnostic tool to determine individual caries activity and risk.^10,23^ Although salivary analysis may provide a general overview of the oral ecology reflecting the caries risk, dental caries is principally a biofilm-induced disease. Viewing this biofilm (dental plaque) as a complex microbial ecosystem has enhanced the understanding of its role in caries development and progression.^15^ Hence, both saliva and plaque samples were analysed for the amounts of *Streptococcus mutans* and *Lactobacillus* in the present study.

The traditional culturing method, one of the most common methods, was used to quantify cariogenic bacteria in plaque and saliva. In this study, the standard plate counting method, expressed in CFU/ml, was used for plaque and saliva bacterial colonies. There was a statistically significant difference in both plaque and salivary bacteria CFUs within all the groups at different time points. However, there was no statistically significant difference between the groups. Similar results were obtained in other clinical studies.^7,26^ The antimicrobial activities of different dentifrices were attributed to their principal ingredients at the time this study was conducted: *Salvadora persica* in the case of herbal dentifrice; kreosotum, *Plantago major* and calendula in the homeopathic dentifrice, and triclosan and fluoride in the conventional dentifrice.

Concerning pH, there was no statistically significant increase in mean salivary pH among all groups at baseline, day 14 and day 28. Similar results were obtained in other studies.^7,11,26^ Similar to the study conducted by Wafa et al,^26^ there was a statistically significant difference in plaque pH on the 14th and 28th day among different groups. Group 1 showed the highest increase in plaque pH among all the groups. The increase in pH can be attributed to *Salvadora persica* which may stimulate parotid gland secretion, thereby raising the plaque pH.^24^

A few limitations of the study may be pointed out. Firstly, the subjects work in a dental setting and were more likely to maintain better oral hygiene compared to the general population. Studies targeting the general population or patients with specific oral health problems should be considered. Although the bacterial count showed reductions over one month, the prolonged effect on the bacterial count needs to be checked, as well as whether the reduced count is stabilised.

## Conclusion

With the data obtained in this study, it can be concluded that the herbal, homeopathic and conventional dentifrices exhibited similar antimicrobial properties by bringing about significant reduction in *Streptococcus mutans* and *Lactobacillus* colony counts. Among all the preventive modalities, herbal dentifrice showed better anticariogenic potential by increasing plaque pH; however, its good anticariogenic potential might be counteracted by the lack of fluoride.
